# Transcriptional profiling of bud dormancy induction and release in oak by next-generation sequencing

**DOI:** 10.1186/1471-2164-14-236

**Published:** 2013-04-10

**Authors:** Saneyoshi Ueno, Christophe Klopp, Jean Charles Leplé, Jérémy Derory, Céline Noirot, Valérie Léger, Elodie Prince, Antoine Kremer, Christophe Plomion, Grégoire Le Provost

**Affiliations:** 1 Forestry and Forest Products Research Institute, Department of Forest Genetics, Tree Genetics Laboratory, 1 Matsunosato, Tsukuba, Ibaraki 305-8687 Japan; 2 INRA, UMR 1202 BIOGECO, F-33610 Cestas, France; 3 Univ. Bordeaux, BIOGECO, UMR 1202, F-33400 Talence, France; 4 Plateforme bioinformatique Genotoul, UR875 Biométrie et Intelligence Artificielle, INRA, Castanet-Tolosan 31326 France; 5 INRA, UR0588 Amélioration Génétique et Physiologie Forestières, Orléans F-45075 France

## Abstract

**Background:**

In temperate regions, the time lag between vegetative bud burst and bud set determines the duration of the growing season of trees (i.e. the duration of wood biomass production). Dormancy, the period during which the plant is not growing, allows trees to avoid cold injury resulting from exposure to low temperatures. An understanding of the molecular machinery controlling the shift between these two phenological states is of key importance in the context of climatic change. The objective of this study was to identify genes upregulated during endo- and ecodormancy, the two main stages of bud dormancy. Sessile oak is a widely distributed European white oak species. A forcing test on young trees was first carried out to identify the period most likely to correspond to these two stages. Total RNA was then extracted from apical buds displaying endo- and ecodormancy. This RNA was used for the generation of cDNA libraries, and in-depth transcriptome characterization was performed with 454 FLX pyrosequencing technology.

**Results:**

Pyrosequencing produced a total of 495,915 reads. The data were cleaned, duplicated reads removed, and sequences were mapped onto the oak UniGene data. Digital gene expression analysis was performed, with both *R* statistics and the R-Bioconductor packages (edgeR and DESeq), on 6,471 contigs with read numbers ≥ 5 within any contigs. The number of sequences displaying significant differences in expression level (read abundance) between endo- and ecodormancy conditions ranged from 75 to 161, depending on the algorithm used. 13 genes displaying significant differences between conditions were selected for further analysis, and 11 of these genes, including those for glutathione-S-transferase (GST) and dehydrin xero2 (XERO2) were validated by quantitative PCR.

**Conclusions:**

The identification and functional annotation of differentially expressed genes involved in the “response to abscisic acid”, “response to cold stress” and “response to oxidative stress” categories constitutes a major step towards characterization of the molecular network underlying vegetative bud dormancy, an important life history trait of long-lived organisms.

## Background

In temperate zones, woody plants go through seasonal cycles with two stages: a growing period when environmental conditions are favorable and a period of non growth in winter (reviewed in [1]). This second period of non growth is known as dormancy, and is defined as the time between bud set in the fall and bud burst in the spring. Dormancy allows trees to survive unfavorable conditions in winter [2] and can be divided into two phases of variable duration and intensity [3]: endodormancy and ecodormancy.

i.) firstly endodormancy: the deepest state of dormancy, in which bud burst is prevented by endogenous factors specific to the meristem. The strength of endodormancy increases from the end of summer, throughout the fall, generally peaking somewhere between the end of October and the end of November in the northern hemisphere depending on the species and the climatic conditions prevailing during the fall [4]. A dormancy breaking test, or forcing test, is the method of choice for assessing the depth of endodormancy in trees [5]. The signals leading to the breaking of endodormancy in perennials are well characterized, temperature playing a major role in both the induction and release of endodormancy [6].

ii.) secondly ecodormancy: this dormancy state occurs when unfavorable external environmental factors (usually temperature in the case of perennials growing in temperate climates) prevent bud burst in late winter and early spring.

The growing season of trees extends from bud burst in the spring to bud set in the fall. This phenological cycle has been shown to be strongly affected by climatic change, higher temperatures having a negative impact on endodormancy (if the requirement for chilling is not fulfilled, then dormancy break is delayed) and a positive impact on ecodormancy (acceleration of bud cell growth) [7]. On the other hand, Kalcsits et al. [8] reported that high temperatures during endodormancy induction in poplar leads to deeper endodormancy and delayed bud burst in the spring [8].

An overall increase in the duration of the growing season has been reported in temperate regions, for both fruit and forest trees (reviewed in [9,10]). In this context, early bud break is a particularly crucial issue in terms not only of the increase in length of the growing season, but also the risk of frost damage [11].

Several quantitative genetic approaches have been used to study the genetic basis of bud phenology in forest trees, focusing essentially on the date of bud burst. Medium to high heritability values have been reported, from 0.43 in *Salix*[12] to 0.98 in *Populus*[13]. Saintagne *et al*. [14] reported repeatability values of 0.15 to 0.52 in pedunculate oak (*Quercus robur* L.), indicating moderate to medium genetic control for this trait, whereas very high heritability values were reported in progeny tests and parent-offspring comparisons for *Quercus petraea*. Derory *et al*. [15] reported the identification of 19 QTLs for bud burst for 13 year × site seasonal observations in a cloned full-sib oak pedigree, suggesting polygenic control of this adaptive trait.

However, the molecular mechanism underlying the regulation of bud dormancy in forest trees remains poorly understood. Most investigations of gene expression have been carried out in model plants, such as *Arabidopsis thaliana,* for which functional candidate genes have been identified [16,17]. These studies highlight the role of phytochromes as central players in day-length sensing. Allona *et al*. [18] reported that dormancy induction in forest trees was driven by molecular mechanisms similar to those involved in the photoperiodic control of flowering time in annuals [19]. However, the mechanisms responsible for the maintenance of dormancy, once it has been established, are also poorly understood. Several molecular studies in perennials, such as kiwi [20], grape [21], potato [22], raspberry [23] and leafy spurge [24], have shown that dormancy signals affect multiple physiological processes, including cell division, secondary metabolism (flavone biosynthesis), hormone signalling (with significant changes in genes responsive to ethylene, auxin, ABA and GA) and oxidative stress. These studies also reported changes in the accumulation of transcripts from many regulatory genes thought to be involved in dormancy transition or regulating specific metabolic pathways involved in dormancy transition. In forest trees, the use of transcriptomic approaches to detect the expression of candidate genes thought to be involved in vegetative bud dormancy remains limited. Derory *et al*. [25] and Rohde *et al*. [17] used targeted approaches (subtractive suppressive hybridization and cDNA-AFLP, respectively) to identify genes displaying differential patterns of expression at different stages of dormancy (induction, maintenance and release). Ruttink *et al*. [26] used high-density microarrays to reveal the existence of gene networks involved in apical bud formation and dormancy induction in poplar. Transgenic studies have also shed light on the molecular mechanisms underlying bud phenology. In comparisons with the peach ever-growing (*evg*) mutant, for example, Jimérez *et al*. [27] identified 23 genes that were upregulated in the wild type, and therefore potentially involved in photoperiod response, growth cessation and the induction of dormancy. In poplar, Ibañez *et al*. [16] reported a potential role of circadian clock-related genes in cold hardiness and delayed bud burst.

In this context, the objective of this study was to characterize the transcriptome of endo- and ecodormant sessile oak (*Quercus petraea*) by whole-transcriptome shotgun sequencing (WTSS or RNA-seq) and to identify genes displaying differential expression between the two phenological stages, with the aim of improving our understanding of dormancy at the molecular level. Firstly, four cDNA libraries were built from total RNA extracted from apical buds harvested during endo- or ecodormancy, from two populations with different bud burst dates (early vs. late flushing populations). The transcriptome was then characterized by pyrosequencing on a Roche 454 FLX platform. In total, 495,915 reads were produced. Reads were mapped onto the oak UniGene sequences generated from 34 cDNA libraries [28]. Digital expression analysis and quantitative PCR identified a set of genes displaying differential regulation between these two developmental phases and possibly inter connected with a gene network. None of these genes was associated with a specific population.

## Results

### Identification of the endodormancy and ecodormancy developmental phases

**Figure 1:**
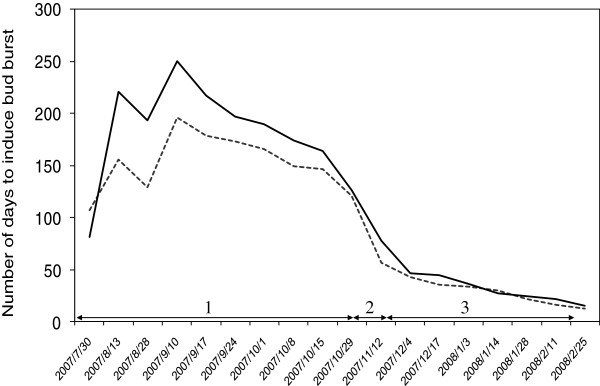
**Variation of apical bud dormancy.** This chart shows the number of days required to induce bud burst in forcing conditions. The dotted line corresponds to the Longchamp population characterized as late flushing in a common garden experiment and the solid line corresponds to the Saint-Jean population (early flushing). Abbreviations correspond to: (1) endodormancy, (2) transition phase and (3) ecodormancy.

The three main developmental phases typically involved in dormancy in perennial plants (for review see [1]) were identified in the two oak populations (Figure 1). The endodormancy phase extended roughly from July 30^th^ 2007 to October 29^th^ 2007, in both populations. Endodormancy levels peaked on the same date in both populations (September 10^th^). A transition phase occurred from October 29^th^ to November 26^th^. Finally, the ecodormancy phase began on December 4^th^.

### Dormant bud transcriptome

**Figure 2:**
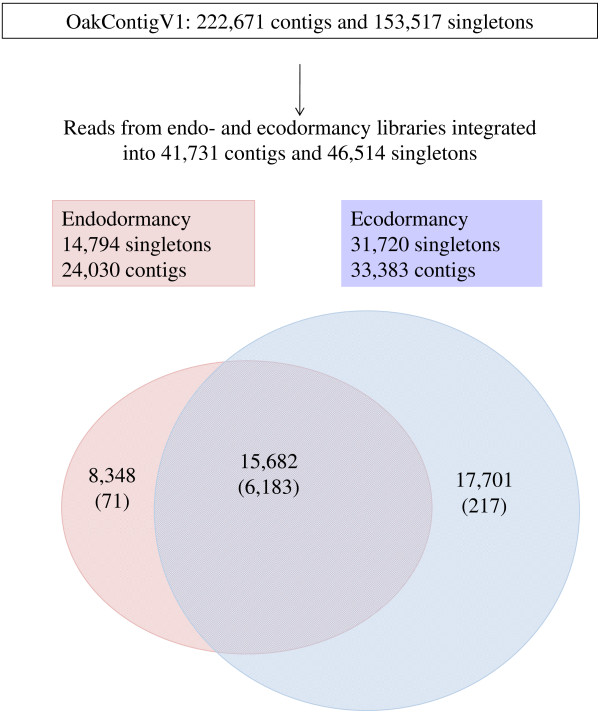
**Assembly and mapping results for endo- and ecodormancy libraries.** In total, 15,682 contigs matched both endo- and ecodormancy reads, whereas 8,348 and 17,701 contigs consisted of only endodormancy and only ecodormancy reads, respectively. Contigs with reads ≥ 5 are indicated in brackets.

A total of 495,915 reads were obtained from the four libraries (Table 1). The cleaning process lead us to eliminate 143,528 (29.9%) reads, resulting in 352,397 (71.1%) reads which were then assembled into OakContigV1 [28]. Reads from the two endodormancy libraries were distributed into 24,030 contigs and 14,794 singletons, whereas those from the two ecodormancy libraries were distributed into 33,383 contigs and 31,720 singletons (Table 1 and Figure 2). Differential expression was analyzed for 6,471 contigs with corrected read numbers ≥ 5 (71, 217 and 6,183 for the endodormancy, ecodormancy and both dormancy libraries, respectively).

**Table 1:** 454-pyrosequencing and assembly statistics for endo- and ecodormancy libraries

	**Endodormancy**		**Ecodormancy**		**Total**
	**LC1**	**SJ1**	**LC2**	**SJ2**	
SRA accession number	SRX019071	SRX019073	SRX019072	SRX019075	
Number of reads	115,050	79,345	137,380	164,140	495,915
Number of reads passed to assembly	70,019	44,732	98,725	138,921	352,397
No. singletons in OakContiV1 from each library (a)	9,937	4,857	15,789	15,931	46,514
No. contigs in OakContigV1 within which reads are assembled (b)	24,030		33,383		41,731*
No. unigene elements in OakContigV1 (a)+(b)	38,824		65,103		88,245*

* The values are not a simple sum of those for endo- and ecodormancy, because there were common contigs to which reads from both libraries were mapped.

### Detection and analysis of differentially expressed genes

**Figure 3:**
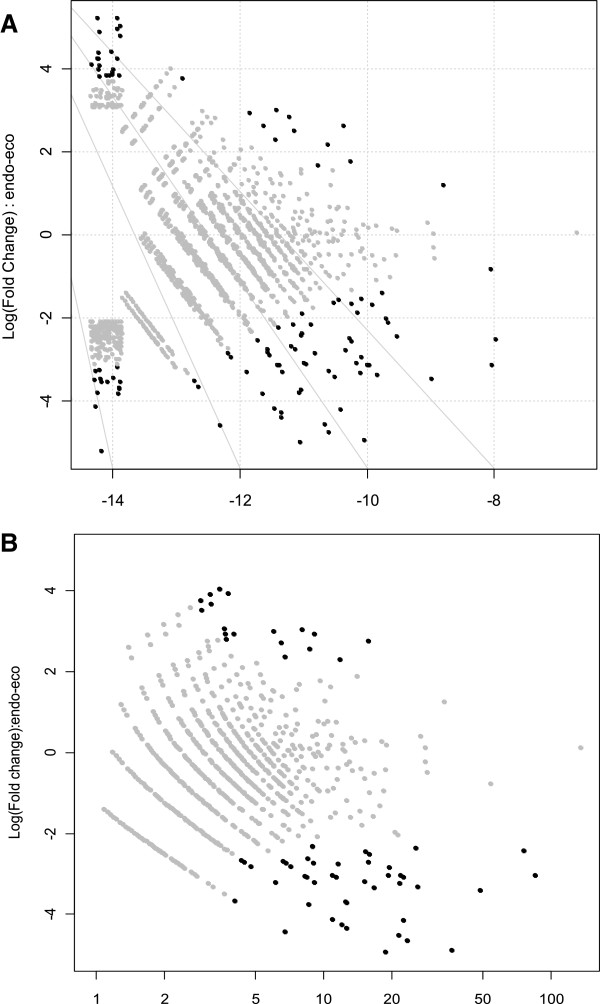
**MA plots for endo- and ecodormancy libraries.** Fold-changes of read counts between endodormancy and ecodormancy are plotted against relative read abundance. Black dots indicate statistically significant contigs displaying differential expression between endo- and ecodormancy libraries as identified with the edgeR (**A**) and DESeq (**B**) packages, respectively. If the number of corrected read counts was zero for either endo- or ecodormancy libraries, no dots were generated for (**B**).

**Figure 4:**
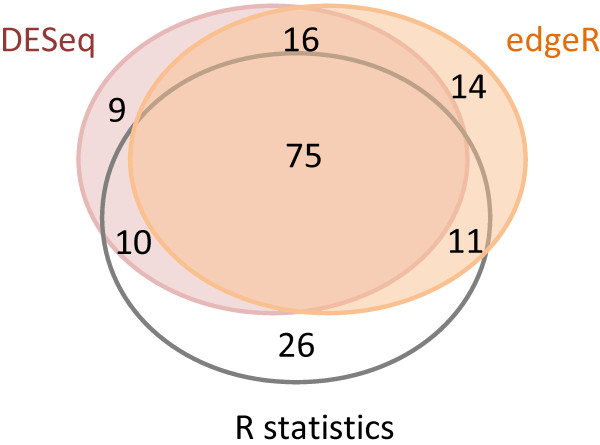
**Venn diagram of differentially expressed genes detected by *R *statistics (white), edgeR (orange) and DESeq (pink).** 122, 73 and 59 contigs were identified as differentially expressed by *R* statistics [29], edgeR [30] and DESeq [31], respectively.

An analysis of differential expression with *R* statistics [29] resulted in 122 (1.89%) contigs (with *R* > 8), suggesting significant differences in expression between the four libraries. The number of contigs for a given value of the *R* statistic was plotted as a function of *R* (see Additional file 1: Figure S1). No genes displaying significant differential expression between populations were identified, allowing us to treat these two populations as biological replicates. edgeR [30] and DESeq [31] R Bioconductor packages detected 116 (1.8%) and 110 (1.7%) contigs, respectively, displaying significant differential expression between endodormancy and ecodormancy (FDR 20%). These values were higher than those using a 5% FDR (59 (0.91%) and 73 (1.13%) contigs identified as significantly differentially expressed by EdgeR and DESeq, respectively). The differences in relative expression between the endo- and ecodormancy libraries and relative read numbers for each contig are displayed as MA plots (smear-plot) in Figure 3. A Venn diagram displaying the number of differentially expressed genes identified by each of the three programs (*R* statistics, edgeR and DESeq) is shown in Figure 4, where 75 contigs were identified by all three programs (MN set), and 161 contigs (MX set) were detected in total. For 26 contigs, only *R* statistics were consistent with differential expression, whereas only 14 contigs were identified as differentially expressed by edgeR and only nine were identified as differentially expressed by DESeq package alone. Heat maps were drawn for both the MX and MN sets (see Additional file 2: Figure S2).

At least one blast hit against SWISS-PROT was found for 54 (33.5%) contigs and at least one blast hit against TAIR9 was found for 102 (63.4%) contigs (see Additional file 3: Table S1). In total, 96 GO terms were assigned to 45 (28.0%) contigs. These GO terms were mapped against 51 GO-slim terms.

Enrichment analysis for AraCyc Pathways and Gene Ontology Groups (EAPG) was then performed both for the gene up-regulated during endo- (i.e. 34 contigs) and ecodormancy (i.e. 41 contigs) identified by the three programs.

Among ecodormancy regulated genes EAPG enabled us to identify three pathways from Aracyc and 44 groups from the GO commons (see Additional file 3: Table S2). Looking to the AraCyc pathway, EAPG found three pathways enriched: Stachyose biosynthesis, oxidative ethanol degradation and beta alanine biosynthesis. EAPG identified also 44 GO groups enriched for ecodormancy up-regulated genes. The first five groups were linked to response to cold, high light intensity, water deprivation, abscisic acid stimulus and water channel activity.

**Figure 5:**
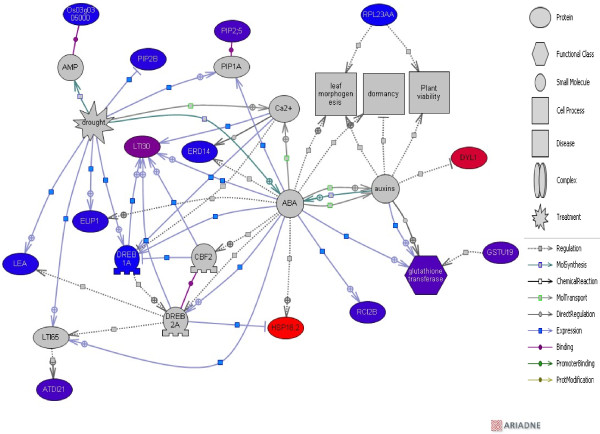
Functional network predicted from the genes upregulated during ecodormancy.

Sub-network enrichment analysis (FNSE) was performed and identified central hubs and their associated partners. Among ecodormancy up-regulated genes three gene-linked hubs (*DREB2A*, *CBF2* and *DREB1A*), three processes- linked hubs (drought, cold and heat shock) and two molecule-linked hubs (ABA and CA^2+^) were identified (see Additional file 3: Table S3 and Figure 5).

The same analysis was also performed for endodormancy up-regulated genes. EAPG identified one AraCyc enriched pathway (cuticular wax biosynthesis) and 39 enriched GO groups (see Additional file 3: Table S4). The first five group based on their p-value were associated with metal ion binding, cellular transition metal ion homeostasis, fatty acid binding, 2-iron2-sulfur cluster binding and epidermis morphogenesis. No central hubs were identified through the FNSE analysis.

### Tissue specificity of differentially expressed genes

Significant differential expression of genes in the MN set was detected for 21 genes (see Additional file 4: Table S5) in an *in silico* expression analysis of cDNA libraries for *Quercus* (see Additional file 5: Table S6). The number of reads mapping onto three genes was positive for bud tissue, but zero for other tissue types (root, xylem and leaf tissue). Two of these UniGene elements (F0SUT5C01ARKJ3.l.qr.1 and F0SUT5C01A7647.1.qr.1) were identified as up-regulated during endodormancy and ecodormancy, respectively, displayed no similarity to any other protein, whereas the third one (F0SUT5C01BB2R7.l.qr.1), was found to be similar to the glutathione S-transferase TAU 19.

### Validation of candidate genes displaying differential expression, by quantitative PCR

Among the five control genes analysed (actin, tubulin β, ubiquitin, ribosomal protein 18S and elongation factor α), only one (ubiquitin) displayed a multi-banding pattern after PCR amplification. Two other genes (actin and tubulin β) displayed changes in expression over the time course of the experiment, in both populations, and were therefore discarded from the analysis. The two remaining genes (ribosomal protein 18S and elongation factor α) were found to display stable expression in both populations, during both dormancy periods, and were therefore used to normalize qPCR data (see Additional file 6: Table S7).

Among the 12 candidate gene analysed, only one encoding a cold-shock protein, was validated in a single population (LC), the 11 remaining genes displayed expression profiles similar to those observed in the 454 sequencing experiment in both populations. Nine of the validated genes were overexpressed during ecodormancy (gibberellin-regulated GASA family protein (*GASA*), glutathione-S-transferase (*GST*), dormancy-associated protein (*DRM1*), dehydrin xero2 (*XERO2*), plasma membrane-intrinsic protein (*PIP*), AWPM-19-like membrane family protein (*AWPM*), gibberellin receptor GID1L2 (*GID*), late embryogenesis abundant protein Lea5 (*LEA*) and early light-induced protein (*ELP*)). The two remaining genes (*basic 7S globulin 2* and *xyloglucan endotransglucosylase/hydrolase9*) were upregulated during the endodormancy period. The expression profiles of the genes analyzed by qPCR are provided in Additional file 7: Figure S3. For reference and candidate genes, PCR efficiency ranged from 87% (basic 7S globulin 2) to 104% (xyloglucan endotransglycosylase/hydrolase9), which fits with the correct functioning of *Taq* polymerase.

## Discussion

Given the extremely high rate of validation of the *in silico* analysis by qPCR, we will consider for the scope of the discussion the core set of 75 contigs (MN set) showing differential expression with three different algorithms (*R* statistics, edgeR and DESeq). No annotation was available for 22 of these 75 contigs (29.3%). A molecular function was attributed to the 53 remaining contigs (see Additional file 3: Table S1).

Gene ontology analysis showed that this set of genes was enriched in genes responsible for abiotic (i.e. cold) and endogenous stimuli (i.e. ABA) and stress-related genes. Similar trends were observed in analyses of gene expression in other plants. In raspberry, the set of genes displaying differential expression during endodormancy and paradormancy included a high proportion of stress-response/defense/detoxification-related genes [23]. In grape, genes relating to oxidative processes and stress responses within the cell are expressed at the end of dormancy [32].

### Genes upregulated during endodormancy

Plants are sessile and must therefore be able to prepare themselves to survive unfavorable conditions. In temperate regions, deciduous trees stop growing, lose their leaves in the fall and enter a state of dormancy to allow them to survive the winter. The detailed molecular mechanisms underlying dormancy induction remain unclear, although an analogy with the mechanisms of vernalization and flowering has been suggested [6]. However, some common features of dormancy in buds, seeds and tubers may help to unravel the underlying molecular mechanisms, although some features are specific [1].

Our transcriptomics analysis revealed that 34 genes in the core (MN) set were overexpressed during endodormancy. Among these genes, two were quantified and validated by qPCR. The first gene encodes a *basic 7S globulin 2* gene (see Additional file 7: Figure S3). Globulin is a well known storage protein present in many plant seeds [33,34]. It accumulates during seed maturation and desiccation before dormancy and is consumed during germination. In maize seeds [35], globulin accumulation is triggered by a plant hormone, abscisic acid (ABA), before dormancy. The upregulation of globulin in oak endodormant buds may also be triggered by ABA produced in response to the environmental conditions prevailing in the fall. Short-day conditions or low temperature may initiate ABA biosynthesis, thereby inducing endodormancy and resulting in a cessation of growth, bud set and cold hardening. The accumulation of *globulin* transcripts in endodormant buds in oak may be considered as a response to an abiotic stimulus. Globulin may be used to provide the nutrients required for bud burst, just as seed storage proteins are used for germination [36].

The second gene encodes a *xyloglucan endotransglucosylase/hydrolase 9* (*XTH*) (see Additional file 7: Figure S3) belonging to the several ontologies (cell wall GO: 0005618, endomenbrane system GO:00012505, hydrolase activity acting on glycosyl bonds GO:00016798 and xyloglucan:xyloglucosyl transferase activity GO:00016762) Over-expression of *XTH* was also reported by Anderson et al. [37] in endo-dormant crown bud of leafy spurge [37]. *XTH* genes constitute a multigene family encoding proteins involved in cell wall construction and disassembly, with diverse levels of expression in *Arabidopsis thaliana*[38]. In tomato, two isozymes of XTH (LeXET and LeXET2) with opposite patterns of expression have been detected [39]. LeXET was abundant in the rapid elongation region, whereas LeXET2 was more abundant in the mature/non elongating region. Enzymatic analysis of xyloglucan endotransglycosylase activity in *Arabidopsis* showed cold tolerance, with optimal activity for this enzyme at temperatures of 12°C to 18°C, consistent with a potential role in cold acclimation or cold-tolerant growth [40]. The *XTH* gene is also strongly expressed in the dormant cambium of *Populus tremula*, suggesting a role for XTH in cell wall modification during dormancy acquisition [41]. Samples of *Prunus percica* buds released from dormancy are rich in *XTH* mRNA [42], probably due to the remodeling of cell wall structure for resumption of the growth cycle. The abundant expression of the *XTH* gene observed in the oak endodormancy library also suggests a potential role in cold acclimation, through the modification of cell wall structure.

The contigs of known function of the core set upregulated during endodormancy included *MLP423* (GO:0009607, response to abiotic stimulus), *pheophorbide a oxygenase* (PAO, Chlorophyllide *a* oxygenase GO:0010277 and Chloroplast GO:0009507) and *agglutinin*. *MLP423* have been reported to be involved in drought regulation in *Populus*[43], though down-regulated in drought treatment. PAO is an enzyme necessary to break down chlorophyll [44], while *agglutinin* functions as glycan binding in *Castanea crenata*[45].

### Genes upregulated during ecodormancy

Ecodormancy is a short-term phenomenon induced by a prolonged period of cold and long-day conditions, which provide an initial signal triggering a shift from endodormancy [46]. In this section we discuss the genes up-regulated during ecodormancy according to the pathway highlighted through the EAPG and FNSE analysis as well as our qPCR analysis (see Additional file 7: Figure S3). Indeed several pathways known to be regulated during ecodormancy (i.e. response to abscisic acid, response to gibberellin stimulus, response to cold and response to oxidative stress) were differentially regulated in our dataset (see Additional file 3: Table S3 and Figure 5). It should also be borne in mind that because of the low number of contigs, as well as the rather relaxed threshold used to declare a significant differential expressions, the analysis of pathways and networks requires further validation.

### Response to abscisic acid

Abscisic acid (ABA) is an important hormone involved in seed dormancy and germination. Recent studies [47] reported that the interaction between ABA and gibberellin was associated with loss of apical dominance. Several genes belonging to this functional category were illuminated through the EAPG (see Additional file 3: Table S3) and the FNSE (Figure 5). Among these differentially expressed genes, an Early Light Inducible Protein (ELIP) was identified. ELIP protein is a nuclear-encoded chlorophyll a/b binding protein of the thylakoid membranes that is upregulated by illumination [48]. The primary function of this protein is thought to be the prevention of photoinhibition in high light conditions, through the dispersal of absorbed light [49], but ELIP also accumulates in response to various stresses, such as cold [50] and UV-B irradiation, in green leaves [51]. In blueberry floral buds, ELIP mRNA levels are high until the end of February (1,200 chilling units), with maximal levels of expression in December (400 chilling units) and no expression in October (0 chilling units) in the absence of cold acclimation [52]. The endodormancy library was based on samples from September 17th, 24th and October 1st. We therefore speculate that the higher levels of ELIP expression recorded for ecodormancy libraries (LC2 and SJ2) than for endodormancy libraries (LC1 and SJ1) are consistent with the buds in our endodormancy sample not being cold-acclimated and that ELIP was not induced in the LC1 and SJ1 libraries despite the buds being in a state of endodormancy. Derory *et al.*[25] showed that ELIP was more strongly expressed in *Q. petraea* buds in the vegetative growth phase than during ecodormancy, in macroarray experiments [25]. ELIP is induced not only by cold, but also by high light intensity. The primary stresses responsible for the upregulation of ELIP in this study and in the study by Derory et al. [25] may therefore be different.

A low temperature inducible protein LTI30 (Xero 2 or dehydrin xero 2) highly expressed during ecodormancy was also identified. This gene is also regulated by ABA [53]. The LTI30 gene is expressed in *Arabidopsis thaliana* leaves after cold stress [54]. Dehydrins (group 11 family LEA proteins or LEA2) are stress-responsive proteins induced by dehydration in conditions of drought, low temperature, salinity and seed maturation [55]. Dehydrin is upregulated after cold acclimation in blueberry floral buds [52]. In Norway spruce, most dehydrin genes are expressed in ecodormant buds and downregulated towards bud burst [56]. A dehydrin-like protein has also been detected in swelling oak buds [25].

Two Plasma membrane Intrinsic Proteins (*PIP2B, PIP1A*) were identified in our transcriptomic approach. These proteins have been shown to be regulated both by ABA and water stress [57]. PIP constitutes a subfamily of aquaporins, membrane proteins that form channels in the membrane, facilitating the movement of water across membranes. The *PIP* gene is also upregulated during vernalization in grape buds [21]. In raspberry, it has also been shown that aquaporin genes were regulated between the different phase of the dormancy [23].

Among ecodormancy up-regulated genes, we identified a Heat Shock Protein (HSP18.2). HSP18.2 (Figure 5) was one of the most expressed genes. HSP 18.2 is also regulated by ABA [58]. HSP have chaperone activity by maintaining protein in their functional conformation and therefore prevent the degradation of proteins damaged during cold stress.

Finally an ATDI21 protein similar to Late Embryogenesis Abundant protein 5 (LEA5) was found to be over-expressed during ecodormancy*.* LEA5 belongs to a family of LEA originally discovered in cotton seeds during late embryogenesis [59,60]. LEA proteins stabilize cellular components in cells subjected to water deficit, probably because of their hydrophilic properties [55]. LEA may also protect the cells during environmental stresses by lowering water potential, stabilizing membrane structure and scavenging active oxygen species. In *Q. petraea* buds [25], *LEA5* mRNAs were detected during ecodormancy, and this upregulation during ecodormancy was confirmed by qPCR [25]. Similarly, in this study, *LEA5* transcripts were shown, by qPCR, to be upregulated in ecodormant buds (see Additional file 7: Figure S3). By contrast, an expression analysis on raspberry buds [23], revealed that both dehydrin-like and LEA proteins were produced in larger amounts during endodormancy. Moreover, poplar apical buds also express *LEA14-A* principally during the endodormancy induction phase [17]. In leafy spurge, Horvath et al. [61] reported also that dehydration genes were down-regulated following the transition from endo- to ecodormancy [61]. These discrepancies may reflect the diverse functions and expression patterns of LEA proteins in different tissues and during diverse developmental stages [62].

### Response to cold

In temperate region, perennial species are able to increase their freezing tolerance in response to low temperature observed during ecodormancy. This process is called cold acclimation and the coordinated expression of thousand genes to induce physiological and biochemical changes in the buds [reviewed by [63]] is required. Several cold inducible genes have been isolated from plants and although some are regulated by low temperature, most of them are known to response both to ABA and water deprivation suggesting a strong interaction between these pathways during ecodormancy. Our functional analyses (EAPG and FNSE) revealed several genes involved in the response to cold (see Additional file 3: Table S2 and S3). First we identified a RCI2B gene highly similar to a Rare cold inducible protein 2B. This gene belongs to a small multigene family regulated by low temperature and ABA. It was suggested that this genes is involved in the maintenance of membrane integrity triggered by low temperature that reduces water availability. RCI2B genes may interact with other membrane proteins to maintain the water status of the cell [reviewed by [64]].

An ERD14 gene highly similar to a dehydrin protein was also identified. Dehydrin proteins are a group of proteins with a protective role against stresses. These proteins are synthesized in response to low temperature and ABA. A positive correlation between dehydrin content (i.e. mRNAs and proteins) and freezing tolerance has been reported in *Fragaria* [Reviewed by [65]]. We could hypothesize that the up-regulation of this genes during ecodormancy is associated with a protective role against low temperature observed in late winter.

Finally, a DREB1A genes over-expressed during ecodormancy was identified. Several studies reported that DREB transcription factors are essential for dormancy regulation in plants [47,66]. DREB1A transcription factor contains an AP2/EREB motif and have a binding function to the promoter containing the CRT/DRE (Dehydratation responsive element) which exists in many LEA genes including ERD genes. Over-expression of DREB1A transcription factors induces in normal condition the over-expression of these cold-stress related genes and confers an improved tolerance to both water stress and low temperature [67]. Over-expression of DREB1A transcription factor during ecodormancy may be interpreted in light to the over-expression observed for LEA5 and ERD14 genes allowing the bud to face low temperature observed during ecodormancy.

### Response to gibberellin stimulus

Gibberellins (GAs) are plant hormones that regulate growth and influence various developmental processes, including stem elongation, germination and dormancy. Two genes belong directly to this functional category (T30D6.7/ GASA, and ERD14), and one gene indirectly involved was also identified and validated by qPCR (GID1). ERD 14 encodes an early response to dehydration protein (see above). Briefly, ERD proteins belong to a multigene family known to be strongly regulated during dormancy in leafy spurge [68]. This protein accumulates during dehydration and is probably involved in the protection of the membrane.

GASA (gibberellin-regulated gene family from *Arabidopsis thaliana*) [69] was originally identified in a gibberellin-deficit mutant of tomato, as a GA-regulated gene [70]. This gene is expressed in a tissue-specific manner [71,72] and is involved in several developmental processes, such as seed germination, stem elongation and flowering. GA regulates growth after the release of dormancy. In potato tubers, endogenous GA content increases when sprouts begin to grow [73] and GA biosynthesis is controlled by the photoperiod [74].

Finally we identified a GID1 encoding gene (probable GA receptor GID1L2) overexpressed during ecodormancy. GID1 is a soluble protein that interacts directly with GA, triggering the gibberellin response [75]. Gibberellic acids are key hormone involved in dormancy, several authors reported that they could replace chilling of dormant buds (reviewed in [76] ). In a microarray analysis of grape buds, GID1 was found to be upregulated during vernalization [21]. On the other hand, a down regulation of the GIB1B gene was observed in endodormant buds of leafy spurge [68]. These authors hypothesized that down regulation of the GIB1B gene in conjunction with DREBs (see above “response to cold”) played a significant role in endodormancy release or ecodormancy maintenance.

### Response to oxidative stress

Three genes (AT3G10020, RPL23AA and GSTU19) belonged to this GO term. GSTU19 is similar to a glutathione-S- transferase (GST). *GST*s are found in several animal and plant cytosols, and have diverse functions in detoxification, oxidative stress resistance and light signaling [77]. In some woody plants, *GST* is upregulated during the release of endodormancy, probably due to the response to oxidative stress [23,78,79] induced by low temperature [80].

### Other genes identified

The *DYL1* (or DRM1 dormancy-associated protein involved in response to fructose stimulus) was up-regulated during ecodormancy. DYL1 is abundantly expressed in axillary buds of pea, with mRNA levels decreasing after decapitation; it is therefore considered a good marker of dormancy [81]. In blueberry buds, *DRM* mRNA levels were highest in a sample collected on December 8th, subsequently decreasing towards bud burst [82]. In grape buds, *DRM* gene expression also decreases progressively towards bud burst, this gene being more strongly expressed during dormancy [32].

Finally, in this study, the expression profile of the *AWPM* (AWPM-19-like membrane family protein) gene was also analyzed by qPCR. AWPM, a membrane protein that confers cold tolerance, has a sequence similar to that of LEA and is induced by ABA [83]. Quantitative PCR showed also upregulation of the cold-shock protein only in the LC2 library (ecodormant apical bud library from the Longchamp population). This protein has been also detected in plants subjected to cold stress for acclimation [84]. The protein binds to nucleic acids and destabilizes RNA structure, thereby affecting protein translation. These results strengthen once again that ABA and cold acclimation are probably the most important pathways involved in ecodormancy regulation.

## Conclusions

The timing of bud burst affects the fitness of individual trees. Bud dormancy changes in nature from the fall to the spring. We investigated the molecular basis of dormancy, by analyzing differences in gene expression between two dormancy phases (endodormancy and ecodormancy) in sessile oak (*Quercus petraea* (Matt.) Liebl.) buds by RNA-Seq. We identified 75 UniGene elements displaying differential expression (in terms of read abundance) between the endo- and ecodormancy phases, with a false discovery rate of 20%. Thirty four contigs were over–expressed during endodormancy and 41 were up-regulated during ecodormancy. Enrichement anlysis revealed that the molecular mechanisms involved in dormancy regulation were different between the 2 conditions. Indeed, enriched GO groups for response to cold, high light intensity and ABA stimulus were found only during ecodormancy while other GO groups - metal ion binding, fatty acid binding and epidermis morphogenesis - were specific for endodormancy.

In conclusion, these results demonstrate that RNA-Seq technology and the associated statistical methods are suitable for the detection of differentially expressed genes. We identified promising expressional candidate genes for future forward genetics studies. They will serve as a stepping stone to further work on dormancy, particularly as concerns woody plants, for which few data are currently available.

## Methods

### Forcing test and bud sampling

Acorns of sessile oak (*Quercus petraea* (Matt) Lieb.) were collected in 2006 from two populations in northern France: Longchamp (LC: lat. 47° 15' 49“N, long. 05° 18' 37”E) and Saint Jean (SJ: lat. 48° 46' 43“N, long. 06° 43' 42”E), separated by a distance of 150 km. These two populations have repeatedly shown differences in the timing of bud burst in common garden experiments. Bulk harvests of acorns were carried out in the two seed stands in the fall of 2006. The acorns collected were sown, in spring 2007, in 5 l containers filled with a peat and sand mixture (1/1, v/v). Following germination, we obtained 560 seedlings for the SJ population and 468 seedlings for the LC population.

A forcing test was carried out, from July 30^th^ 2007 to February 25^th^ 2008, in a greenhouse at the INRA forestry research station (south-western France). We selected about 25 seedlings from each population each week. Ten seedlings were used for transcriptome analysis. Apical buds were decapitated from these 10 seedlings and immediately frozen in liquid nitrogen. The 15 remaining seedlings were transferred to the greenhouse in the following conditions: 16-hour photoperiod with a daytime temperature of 25°C and a nighttime temperature of 18°C. For each batch of seedlings, the date of bud burst (defined here as swelling buds, see Additional file 8: Figure S4) was evaluated by observations three times per week, making it possible to determine the number of days required to induce bud burst. The phenological curve obtained in the forcing test (Figure 1) was then used to locate the time points at which endo- and ecodormancy could be differentiated in each population. As endodormancy peaked on the same date in each population and we wished to capture the transcripts expressed at endodormancy in both populations, we calculated the mean number of days to bud burst over the endodormancy period for the two populations together (185 days). This number was then used to determine the sampling date for endodormancy and extended the sampling period to one week before and one week after, for library construction. For ecodormancy, apical buds harvested on January 14^th^, January 28^th^ and February 11^th^ 2008 were used to generate the library, because the number of days required to induce bud burst was short in the two populations (16 and 20 days on average for the LC and SJ populations, respectively).

It should be borne in mind that with this forcing test genes identified are related to variations of the dormancy stage or natural environmental variations (mostly light and temperature) or both. However it is not possible to disentangle these effects as light and temperature variations trigger the shift from endo- to ecodormancy.

### Total RNA extraction and 454-pyrosequencing

Total RNA were extracted as described by Le Provost *et al.*[85].

**Figure 6:**
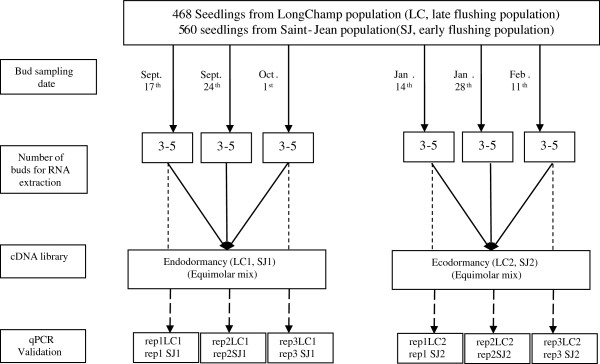
Sampling scheme for cDNA library construction and qPCR analysis for seedlings from two populations (Longchamp and Saint Jean).

RNA quantity and quality were then estimated by both spectrophotometry and visual inspection after electrophoresis in 2% agarose gels. We eliminated any residual DNA, by incubating the RNA with RNase-free DNase RQ1 (Promega, Madisson WI, USA). We used two different strategies to quantify transcript accumulation. (1) Before 454-sequencing, we established two cDNA libraries (corresponding to endodormancy and ecodormancy) for each of the two populations (LC and SJ), with the SMART PCR cDNA synthesis kit (Clontech, Palo Alto, CA, USA), according to the manufacturer’s instructions. Total RNA was extracted independently from apical buds harvested on September 17^th^, 24^th^ and October 1^st^ for endodormancy and on January 14^th^, 28^th^ and February 11^th^ for ecodormancy, and equimolar mixtures were then established, to obtain a representative library for each population and developmental stage (Figure 6). (2) For reverse transcription and quantitative PCR (qPCR), the same RNA samples were used, but without pooling, so as to obtain three biological replicates for each dormancy stage and population. Each library was bar-coded with MID primers and run on a Roche 454 GS-FLX platform. Reads from the four libraries are accessible at **SRA (Short Read Archive) of NCBI** (http://www.ncbi.nlm.nih.gov/sra).

### Cleaning, assembly and annotation of the dormant bud transcriptome

Reads were extracted with the sfffile program of Roche 454 Life Science software (Branford, CT, USA). Each 454-read was screened by cross_match [86] for primers and adaptors and was then masked. The longest non masked region was extracted and further cleaned up with SeqClean [87]. The shorter regions were discarded to eliminate potential chimeras. Cleaned reads were assembled into OakContigV1 [28], which was constructed with ESTs from 20 Sanger- and 14 454-sequencing libraries, including the four libraries studied here (LC1, LC2, SJ1 and SJ2, Figure 6), with the TGICL pipeline (see [28] for detailed procedures).

### Detection and annotation of differentially expressed genes

We assumed that the read counts corresponding to each contig for each library were indicative of the level of gene expression. We corrected for artificial duplication biases during emulsion PCR, by developing an in-house tool called ‘pyrocleaner’ [88]. We defined duplicated reads as clusters in megablast [89] analyses with a minimum hit score of 100, a percent identity cutoff of 98, with an alignment of reads starting at exactly the same position and ending within 70 bp of the end of the longest sequence. Duplicated reads were not taken into account when counting the number of reads in each contig.

We corrected the number of reads in each contig for the coverage of each read and the length of the corresponding contig (Method II in [90]). We first counted the number of reads for each nucleotide position (position score) in the contig, for each library. A total score was obtained by summing the position scores over the entire contig for each library. This total score was divided by the length (base pairs) of the contig. The resulting values were rounded off for use as relative levels of gene expression (Cval: corrected value) for each library. Contigs with a total Cval over four libraries ≥ 5 were used for further analysis. We used three statistical methods to determine the significance of differences in gene expression between endo- and ecodormancy libraries. In the first method, *R* statistics [29] were calculated. This method can be used for the comparison of differential expression between multiple cDNA libraries. Contigs with *R* > 8 were considered statistically significant. We also used two R Bioconductor packages: edgeR [30] and DESeq [31]. We applied a false discovery rate (FDR) of 0.2 as a type I error for significant differential expression. The genes identified as differentially expressed with the three methods were combined, to define a minimum (MN) and a maximum (MX) set of differentially expressed genes. The MN set was defined as the intersection of the genes identified as differentially expressed by the three programs, whereas the MX set was defined as all the genes identified as differentially expressed by at least one of these techniques. Heat maps were drawn for both the MN and MX sets, for visualization of the relationships between differentially expressed genes by gplots libraries in R [91]. We assessed the statistical significance of GO slim terms for differentially expressed genes, by using the goseq [92] R Bioconductor package to overcome the length bias inherent to RNA-seq data. The FDR was set at 0.05. We used both the MN and MX sets for goseq analysis.

Homology searches were carried out with BlastX [93] against the SWISS-PROT [94] and TAIR9 protein databases. The e-value cutoff for the Blast searches was set at 1 × 10^-5^. Gene ontology (GO) [95] annotations were based on the top Blast hit against SWISS-PROT database. Blast2GO software [96] was used to summarize GO, which were mapped onto plant-specific GO-slim terms.

### Enrichment analyses

Enrichment Analysis for Pathways and groups (EAPG) and Sub-networks (FNSE) for selected genes were performed using Ariadne Pathway Studio 9 Desktop edition Software and the Resnet Plant Version 4 database (Ariadne Genomics Inc., Rockville, MD, USA). As described on Ariadne web site (http://www.ariadnegenomics.com) the “Find Pathways/Groups enriched with selected entities” function (named here as EAPG for simplicity) execute a Fisher’s exact test on each pathway or group and returns informations relative to overlapping entities together with the p-value of the statistical test. EAPG was executed against Gene Ontology as well as AraCyc plant metabolic pathway [97,98]. The “Find sub-networks Enriched with selected entities” function (FSNE) was used to identify the set of entities (sub-networks) organized by specific relationship using Fisher’s Exact Test.

### Detection of bud-specific genes by *in silico* analysis of differentially expressed genes

Eleven cDNA libraries (see Additional file 5: Table S6) constructed from four tissue types (bud, leaf, root and xylem) were used to analyze the bud-specific expression of genes identified as differentially expressed between the endo- and ecodormancy phases (75 contigs in the MN set, 34 of which were upregulated during endodormancy, the other 41 being upregulated during ecodormancy). For a detailed description of these cDNA libraries, see [28]. We counted the number of reads mapping to the MN set from these libraries, with OakContigV1 [28], for each tissue type and used *R* statistics [29] to analyze differential expression between tissue types regarded as virtual cDNA libraries. Values of *R* > 8 were considered to indicate statistically significant differences in expression between tissue types.

### Reverse transcription and quantitative real-time PCR (qPCR)

The following criteria was applied to select candidate genes: (1) first, genes displaying the strongest differential expression in previous statistical analyses were selected, and (2) the molecular function of the genes selected in bud development in other species was also investigated using the ***Arabidopsis *database** available at the following URL (http://www.arabidopsis.org). Using this strategy 13 candidate genes based on expression and function data (see Additional file 6: Table S7) were identified.

Among the 13 genes analyzed, only one encoding an 18.1 kDa class I heat-shock protein, displayed a multi-banding pattern after PCR amplification and was discarded from the analysis. Relative quantification was therefore carried out for the remaining 12 genes.

Five control genes were also selected, for which we analyzed expression profiles in our experimental design.

For quantification, 1 μg of total RNA were reverse transcribed using the Improm-II™ reverse transcription system (Promega®), according to the manufacturer’s instructions. The cDNA generated was diluted by a factor of 10 for qPCR analysis. We performed qPCR and quantification on a Chromo4™ Multicolor Real-Time PCR Detection System (Bio-Rad Laboratories, Inc. Hercules, CA, USA), according to a procedure described elsewhere [99]. PCR primer pairs were designed with Primer3 software [100]. Primers were designed to have an optimal size of 20 bp (18–22 bp), a GC content of 40 to 60%, and a Tm of 60°C. Other criteria, such as primer self-annealing, were also taken into account. Oligonucleotides were synthesized by Eurogentec (Liege, Belgium). Primer pairs are listed in Additional file 6: Table S7.

We performed qPCR and data analysis as previously described [99]. Briefly, data were analyzed with the Excel (Microsoft) macro GENEX v1.10 (Gene Expression Analysis for iCycle iQ® Real-Time PCR Detection System, v1.10, 2004, Bio-Rad Laboratories), using the methods derived from published algorithms [101]. Expression levels were normalized with respect to the stable levels of expression of genes encoding an elongation factor and an 18 S ribosomal protein. GeNorm software was used to assess the stability of the genes used as internal control.

## Competing interest

The authors declare that they have no competing interests.

## Authors’ contributions

SU analyzed the data and drafted the manuscript with GLP, JCL AK and CP. GLP and JD conceived the study. GLP prepared the libraries with VL and performed qPCR with EP. CK and CN carried out the bioinformatic analysis. Pathway Studio analysis was performed by JCL. AK coordinated the project. All the authors read and approved the final manuscript.

## Supplementary Material

Additional file 1: Figure S1 Distribution of *R* statistics. Description of data: *R* was calculated as described by Stekel *et al*. [29]. Differentially expressed contigs are shown in the gray area. Click here for file

Additional file 2: Figure S2 Heat maps for genes displaying differential expression between the endo- and ecodormancy phases. Description of data: A) MN and B) MX sets, as defined in the methods section. Click here for file

Additional file 3: Table S1 Candidate genes displaying differential expression between the endo- and ecodormancy phases. Description of data: Differentially expressed genes were detected by three programs (*R* statistics, edgeR and DESeq). Their annotations with SWISS-PROT and TAIR9 databases and gene ontology were based on the top hit in BLASTX homology searches. 1: Differentially expressed genes (upregulation during ecodormancy) at a false discovery rate of 20%.; -1: differentially expressed genes (upregulation during endodormancy) at a false discovery rate of 20%.; NA: No annotations available. **Table S2.** EAPG results for ecodormancy up-regulated genes. **Table S3.** FNSE results for ecodormancy up-regulated genes. **Table S4.** EAPG results for endodormancy up-regulated genes. Click here for file

Additional file 4: Table S5 Tissue-specific *in silico* expression analysis of genes differentially expressed in the endo- and ecodormancy phases. Description of data: Genes differentially expressed between tissues were identified by *R* statistics. *R* values > 8 were considered statistically significant (cells in yellow). Cells in green indicate contigs to which the reads from the bud library were mapped but to which no reads from other libraries were mapped. Click here for file

Additional file 5: Table S6 cDNA libraries for European oak used for *in silico* analysis of tissue-specific expression. Description of data: The tissue type, the number of total reads in the OakContigV1, the library code used by Ueno *et al.*[28] and the Evoltree library code are given in the table. Click here for file

Additional file 6: Table S7 Primer pairs used for qPCR. Description of data: List of the genes validated by qPCR in our experiment. Abbreviations are as follows: Tm: annealing temperature, NA: not available. Click here for file

Additional file 7: Figure S3 Relative expression profiles of the genes analyzed by qPCR. Description of data: For each dormancy stage, the expression levels of the genes were estimated over three biological replicates. Error bars represent standard deviations (N=3). Abbreviations are as follows: LCendo: Longchamp endodormancy, LCeco: Longchamp ecodormancy, SJendo: Saint-Jean endodormancy and SJeco: Saint-Jean ecodormancy. Click here for file

Additional file 8: Figure S4 Detailed phenological scale of bud development in *Quercus petraea* (Matt) Lieb. Description of data: Stage 0: quiescent bud, scales visible, stage 1: swelling bud, stage 2: bud opening, stage 3: leaves visible, stage 4: internode expansion. Click here for file
