# Supplementary figures and images for: Transcriptional profiling of bud dormancy induction and release in oak by next-generation sequencing
Source: BMC Genomics. 2013 Apr 10;14:236. doi: 10.1186/1471-2164-14-236 (PMC3639946; doi:10.1186/1471-2164-14-236)

## Slide 1
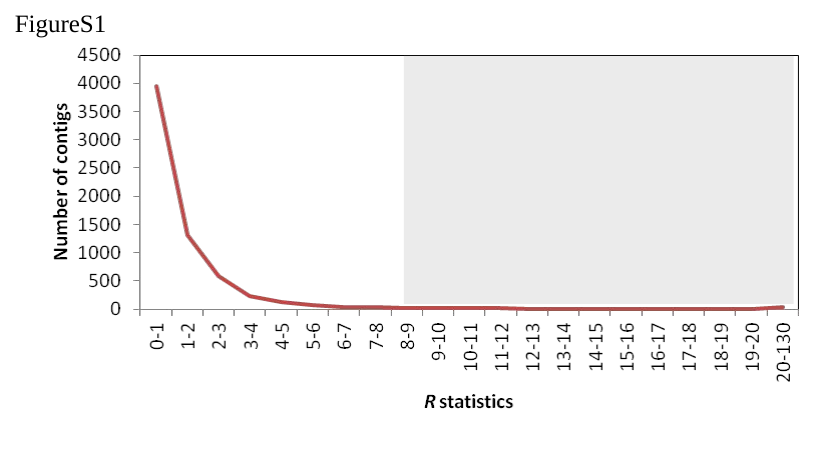

FigureS1

Supplement: Additional file 1: Figure S1 — Distribution of R statistics. Description of data: R was calculated as described by Stekel et al. [29]. Differentially expressed contigs are shown in the gray area. [file 1471-2164-14-236-S1.ppt]

## Slide 1
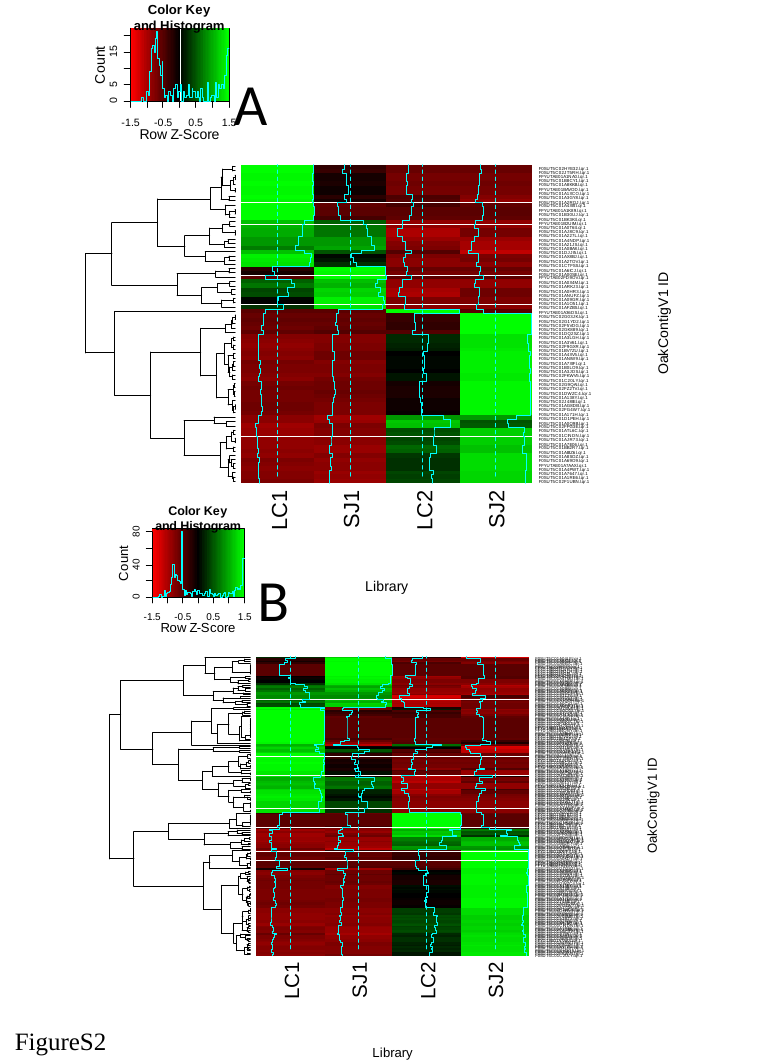

A
B
FigureS2

Supplement: Additional file 2: Figure S2 — Heat maps for genes displaying differential expression between the endo- and ecodormancy phases. Description of data: A) MN and B) MX sets, as defined in the methods section. [file 1471-2164-14-236-S2.ppt]

## Slide 1
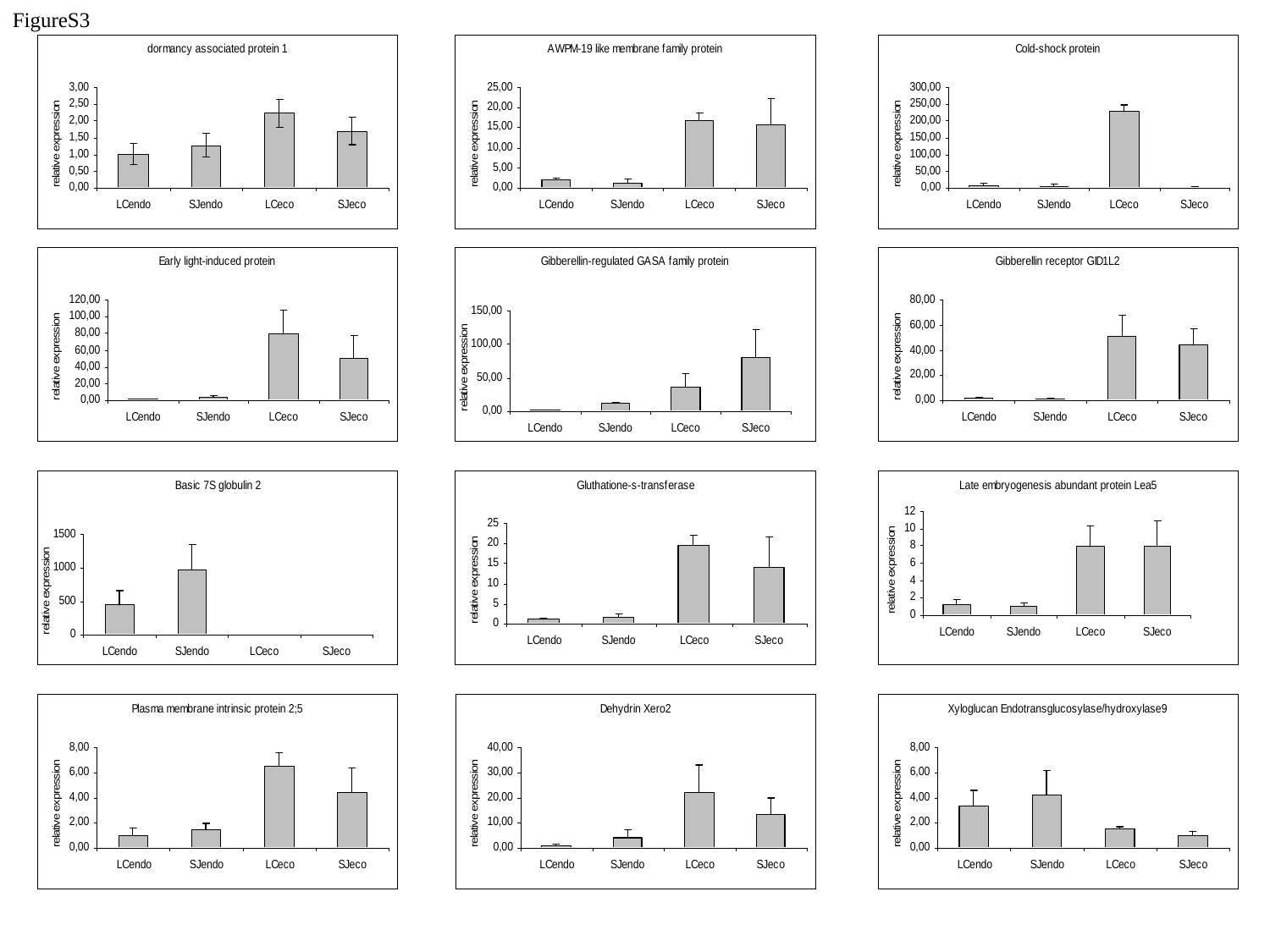

FigureS3

Supplement: Additional file 7: Figure S3 — Relative expression profiles of the genes analyzed by qPCR. Description of data: For each dormancy stage, the expression levels of the genes were estimated over three biological replicates. Error bars represent standard deviations (N=3). Abbreviations are as follows: LCendo: Longchamp endodormancy, LCeco: Longchamp ecodormancy, SJendo: Saint-Jean endodormancy and SJeco: Saint-Jean ecodormancy. [file 1471-2164-14-236-S7.ppt]

## Slide 1
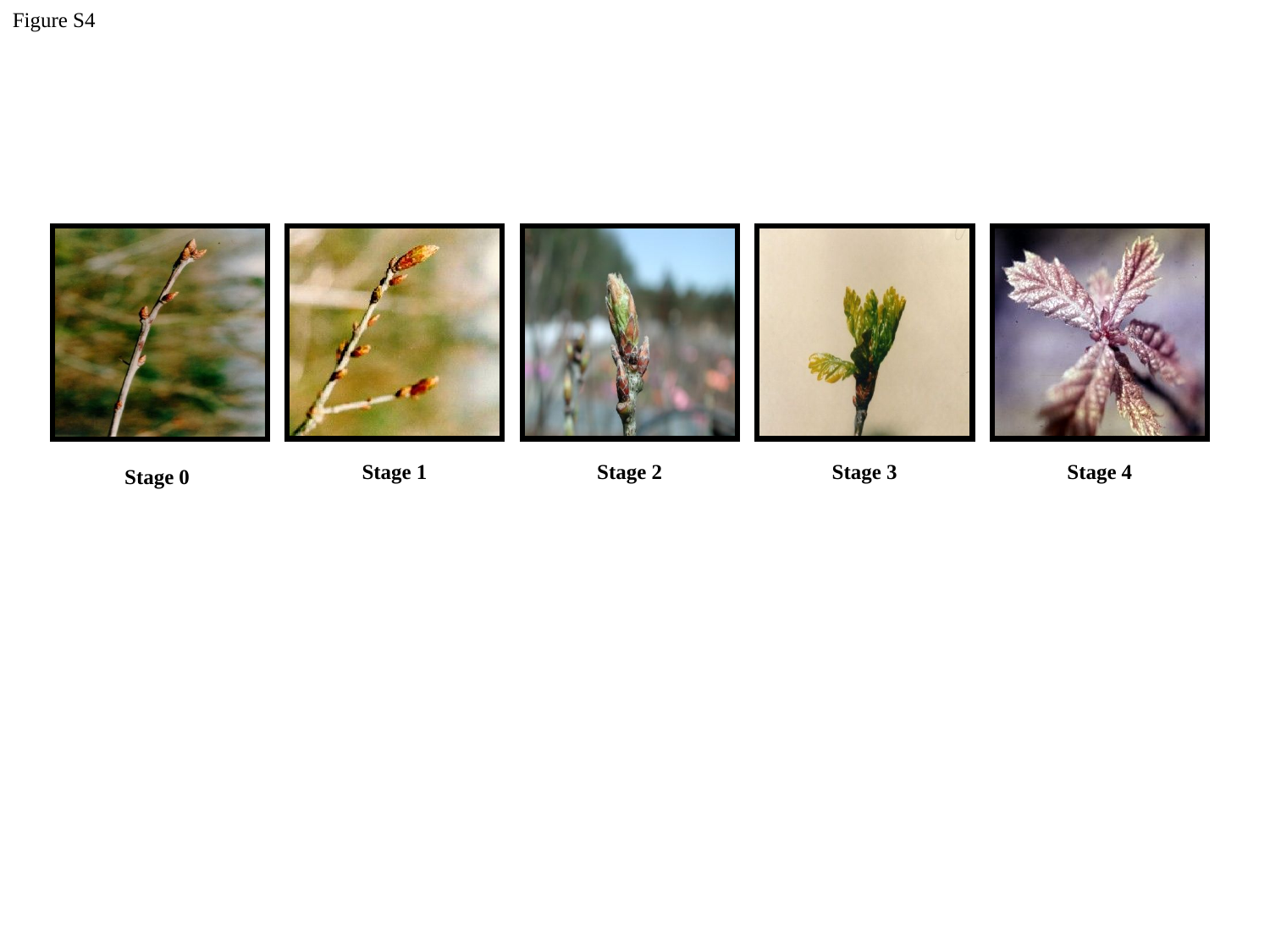

Figure S4
Stage 0
Stage 1
Stage 2
Stage 3
Stage 4

Supplement: Additional file 8: Figure S4 — Detailed phenological scale of bud development in Quercus petraea (Matt) Lieb. Description of data: Stage 0: quiescent bud, scales visible, stage 1: swelling bud, stage 2: bud opening, stage 3: leaves visible, stage 4: internode expansion. [file 1471-2164-14-236-S8.ppt]
